# Decoding Apelin: Its Role in Metabolic Programming, Fetal Growth, and Gestational Complications

**DOI:** 10.3390/children11101270

**Published:** 2024-10-21

**Authors:** Nikolaos Loukas, Dionysios Vrachnis, Nikolaos Antonakopoulos, Sofoklis Stavros, Nikolaos Machairiotis, Alexandros Fotiou, Chryssi Christodoulaki, Markos Lolos, Georgios Maroudias, Anastasios Potiris, Petros Drakakis, Nikolaos Vrachnis

**Affiliations:** 1 Department of Obstetrics and Gynecology, Tzaneio General Hospital, 185 36 Piraeus, Greece; 2 Third Department of Obstetrics and Gynecology, University General Hospital “ATTIKON”, Medical School, National and Kapodistrian University of Athens, 124 62 Athens, Greece; 3 Department of Obstetrics and Gynecology, University of Patras, 265 04 Patras, Greece; 4 Department of Obstetrics and Gynecology, Santorini General Hospital, 847 00 Thira, Greece; 5 Vascular Biology, Molecular and Clinical Sciences Research Institute, St George’s University of London, London SW17 0RE, UK

**Keywords:** apelin, amniotic fluid, fetal development, fetal growth restriction, small for gestational age (SGA), fetal metabolic programming, fetal hypoxia, preeclampsia, insulin

## Abstract

Placental insufficiency and gestational diabetes, which are both serious pregnancy complications, are linked to altered fetal growth, whether restricted or excessive, and result in metabolic dysfunction, hypoxic/oxidative injury, and adverse perinatal outcomes. Although much research has been carried out in this field, the underlying pathogenetic mechanisms have not as yet been fully elucidated. Particularly because of the role it plays in cardiovascular performance, glucose metabolism, inflammation, and oxidative stress, the adipokine apelin was recently shown to be a potential regulator of fetal growth and metabolic programming. This review investigated the numerous biological actions of apelin in utero and aimed to shed more light on its role in fetal growth and metabolic programming. The expression of the apelinergic system in a number of tissues indicates its involvement in many physiological mechanisms, including angiogenesis, cell proliferation, energy metabolism, inflammation, and oxidative stress. Moreover, it appears that apelin has a major function in disorders such as diabetes mellitus, fetal growth abnormalities, fetal hypoxia, and preeclampsia. We herein describe in detail the regulatory effects exerted by the adipokine apelin on fetal growth and metabolic programming while stressing the necessity for additional research into the therapeutic potential of apelin and its mechanisms of action in pregnancy-related disorders.

## 1. Introduction

Adipose tissue is a complex organ that exerts pleotropic effects, where it serves not only as fat storage but it also carries out endocrine functions by producing and secreting adipokines, including adiponectin chemerin, leptin, vaspin, omentin-1 nesfatin-1 (nesfatin), visfatin/PBEF/NAMPT, and apelin. Adipokines, which are bioactive polypeptides, modulate appetite, glucose homeostasis, body weight, blood pressure, and inflammation [1]. Apelin, which is chiefly known for its actions on glucose metabolism, is an adipokine that is strongly expressed and released by adipocytes and other tissues. It exerts additional actions related to cardiovascular functions, angiogenesis, cell proliferation, body fluid homeostasis, inflammation, and oxidative stress [2,3].

The fact that pregnancy is a highly dynamic state closely associated with metabolic changes, including hyperlipidemia, decreased insulin sensitivity, and oxidative stress, indicates that apelin most probably plays a key role during this period. Furthermore, apelin was hypothesized to be a mediator of metabolic disorders, such as gestational diabetes. Of note, placental hypoperfusion, impaired function, and hypoxia are thought to be the underlying mechanisms of several pregnancy disorders, e.g., preeclampsia and fetal growth restriction (FGR), and their consequences. The present review aimed to provide an update on apelin actions as regards fetal nutrition and metabolism, as well as on its role in common pathologies of fetal growth and maternal hemodynamics.

## 2. The Apelinergic System: Structure, Signaling, and Metabolic Functions

### 2.1. The Peptide Apelin: Its Production and Receptor Location

Apelin is a 36-amino-acid peptide that was originally purified by Tatemoto et al. in 1998 from bovine stomach extracts. The human pre-proapelin gene is located on chromosome X at locus Xq25-q26.1 and encodes an immature 77-amino-acid prepropeptide, with a signal peptide in the N-terminal region and three exons [4]. The functional diversity of apelin originates from the small variations in the transcription process throughout various tissues, which leads to the presence of transcripts with a range of sizes. Having translocated into the endoplasmic reticulum and cleaved the signal peptide, the 55-amino-acid proprotein generates several active fragments via post-translational processing, which are characterized by a number of amino acids, including apelin-13, apelin-17, apelin-28, and apelin-36 [5,6]. Apelin-13 and apelin-36 are highly active, while apelin-13 is mainly responsible for the biological activities of mature apelin. Splicing by angiotensin-converting enzyme-related carboxypeptidase seems to be the main degradation pathway [7].

Apelin is the ligand of the orphan class A (rhodopsin-like) G-protein-coupled receptor APJ, which is found throughout the human body. Specifically, it is expressed on the surface of many cell types and in several organs, such as adipose tissue, heart, lung, brain, breast, liver, gastrointestinal tract, kidney, adrenal glands, endothelium, human plasma, and placenta [8,9]. The APJ human gene (*APLNR*), whose location is on chromosome 11q12, encodes a seven-transmembrane protein (composed of 380 amino acids) and was first identified in 1993; it shares a close identity with the angiotensin II receptor, as they both have 31% amino acid (aa) sequence homology [10]. Despite their resemblance, angiotensin II is unable to bind to and activate APJ [11].

### 2.2. Apelin: Its Intracellular Signaling Pathways and Its Functions and Actions

APJ can be activated by the different apelin isoforms and interacts with G proteins (Gα, Gβ, and Gγ), which leads to the activation of various signaling pathways. Among the intracellular signaling pathways are protein kinase B (PKB or Akt), extracellular signal-regulated kinases (ERK1/2), and p70S6 kinase, where the specific pathway utilized depends on the tissue and cell type. Apelin also acts as an inhibitor of cAMP production [12,13]. The various roles of apelin are most likely mediated through the different signaling pathways.

The widespread tissue dispersion of the apelin/APJ system (or apelinergic system) suggests that it is implicated in a large number of physiological processes: these include body fluid homeostasis, regulation of energy metabolism, endocrine stress response, cardiac contractility, blood pressure, angiogenesis, and cell proliferation. Apelin is also involved in pathological conditions such as diabetes, obesity, heart failure, and cancer [10,14,15,16].

### 2.3. Apelin: An Adipokine with Inflammation and Oxidation Regulatory Functions

Adipokines, such as apelin, have become the focus of much research regarding their involvement in the metabolic conditions associated with pregnancy [17] since their capacity to affect metabolism throughout this period might potentially be utilized as a target in the context of future therapeutic interventions [18]. Adipokines are, moreover, being investigated today concerning their relationship with several aspects of fetal growth and physiology [19]. Apelin, which is strongly expressed and secreted by adipocytes, is categorized as an adipokine. It has been established that adipose tissue both stores and mobilizes body fat and also acts as a synthesizer of bioactive molecules that control metabolic balance and homeostasis [10]. It is a highly dynamic endocrine organ composed of both white and brown adipose tissue. While white tissue is mainly used for energy storage, brown adipose tissue contributes to adaptive thermogenesis and operates as the main site of non-shivering thermogenesis in response to environmental changes by utilizing free fatty acids. Adipose tissue is characterized by high morphological and functional plasticity. Progenitor cells and mature adipocytes can potentially differentiate into beige (brite) adipocytes, which are cells with thermogenic effects that resemble brown adipocytes; this constitutes a process called the browning of white adipose tissue [20].

It was suggested that apelin contributes to this phenomenon and also induces brown adipogenesis by enhancing the differentiation and activity of brown adipocytes via the PI3K/Akt and AMPK signaling pathways [21]. Apelin production, which is regulated by insulin, was found to be increased during adipocyte differentiation [22]. Literature data reveal that apelin plasma levels and body mass index (BMI) are positively correlated. While obesity may upregulate apelin levels, this occurs only when the condition is associated with hyperinsulinemia; thus, what mainly produces this augmentation in apelin expression appears to be increased insulin [22].

As previously stated, apart from metabolic processes, the apelinergic system is also involved in several physiological processes. Literature data suggest that apelin also has anti-inflammatory effects through inhibiting the release of inflammatory mediators, such as the nuclear factor-κB (NF-κB) pathway, and by upregulating the extracellular signal-regulated kinases 1 and 2 (ERK1/2) pathway [23]. It furthermore suppresses the production and release of reactive oxygen species (ROS) in adipocytes and stimulates the expression of antioxidant enzymes [10]. Inflammation and oxidative stress are the main precursors of tissue damage and multiple disease states. Thus, apelin could also be targeted as a factor to prevent or reverse cellular malfunction and/or damage.

### 2.4. Apelin as a Metabolic Regulator

**Figure 1:**
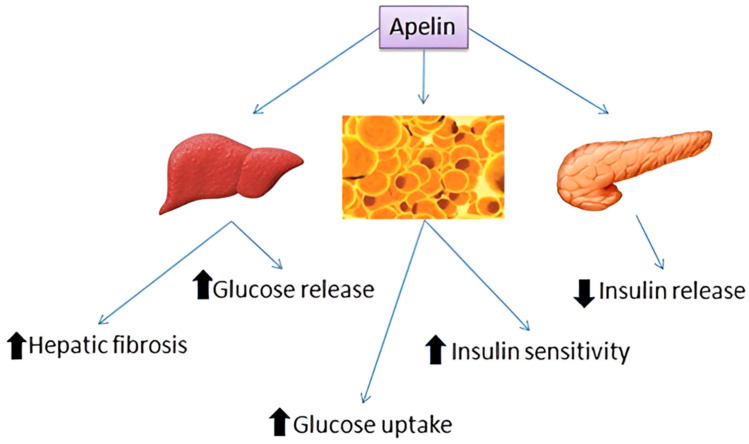
Apelin metabolic functions. Apelin has a pivotal role in energy homeostasis through increasing fatty acid oxidation, lipolysis, and glucose uptake by adipose tissue and skeletal muscles. In addition, it promotes insulin sensitivity in peripheral tissues via inhibiting insulin release from the pancreas via a negative feedback loop. However, in vitro studies showed that glucose-stimulated insulin secretion may be raised by 30% via a high dose of apelin. Apelin can also increase the pancreatic islet cell mass. Its production, as mentioned, is mainly upregulated by insulin. An increase in apelin in states of insulin resistance constitutes a compensatory mechanism of the body, as apelin increases insulin sensitivity. The component of the aperlinergic system that affects processes linked to energy metabolism seems to be mediated via the AMPK/eNOS and PI3K/Akt pathways. In the liver, apelin seems to be associated with hepatic fibrosis. In human and mouse hepatocytes, apelin has controversial effects, as it ameliorates glycogen synthesis, whereas hypothalamic apelin may stimulate liver glycogenolysis and gluconeogenesis [29].

Apelin has a pivotal function in energy metabolism, as it contributes to lipolysis, fatty acid oxidation, adipose tissue angiogenesis, and glucose uptake, and it promotes insulin sensitivity [10,24] (Figure 1). It was established via both human and mouse studies that apelin serum levels are associated with nutritional status, as well as with insulin concentrations in plasma. A number of studies suggested that apelin levels in plasma may be elevated in obesity and type 2 diabetes [25]. Regarding gestational diabetes mellitus, the results are contradictory, where some studies reported an increase in apelin levels in mothers with this complication and others observed no change or even a decrease [26,27,28].

An increase in apelin in the above states of insulin resistance constitutes a compensatory mechanism of the body given that apelin increases insulin sensitivity. Exogenous apelin enhances glucose tolerance, insulin sensitivity, and fatty acid oxidation in these insulin-resistant disorders and simultaneously inhibits glucose-stimulated insulin secretion in pancreatic islets via a negative feedback loop; this action points to an association with glucose homeostasis. An animal study revealed that mice with an apelin deficiency had decreased insulin sensitivity and hyperinsulinemia accompanied by reduced adiponectin levels [10,30]. Adiponectin, which is the most abundantly produced protein synthesized by adipose tissue, plays a key role in metabolism via its reduction in insulin resistance [31] and its anti-inflammatory functions. In a study that evaluated both molecules in pregnant and nonpregnant women, a significant inverse association of apelin and adiponectin was noted, which implies a correlation between these two adipokines [32] that is possibly attributable to their similar actions since both possess insulin-sensitizing and anti-inflammatory properties.

As stated above, numerous studies pointed to the possibility that apelin possesses antidiabetic properties given that it augments glucose uptake and metabolism [33,34]. Apelin’s potent glucose-lowering effect is mediated through elevated glucose uptake in both skeletal muscle and adipose tissue [35]. The administration of apelin improves insulin sensitivity in obesity and diseases related to insulin resistance [33,34,36]. Apelin, which is also expressed in pancreatic islets, although regulated by glucocorticoids, is not regulated by glucose [10]. It is hence evident that apelin is activated in response to biological stress.

**Figure 2:**
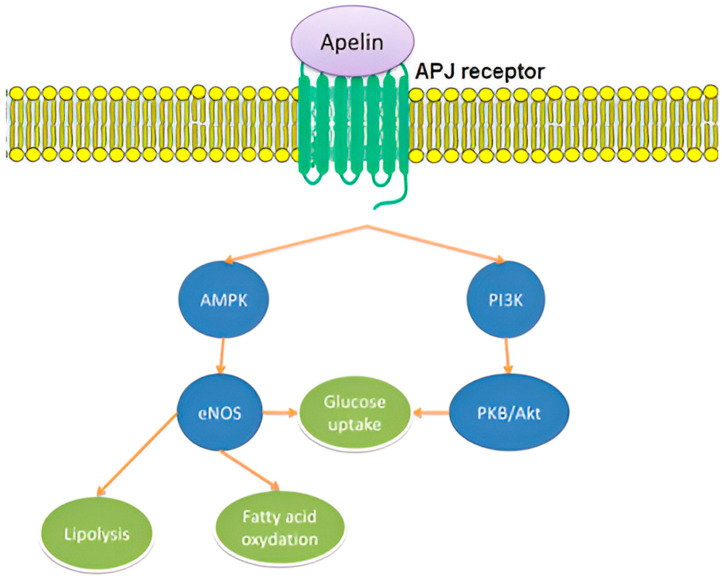
Apelinergic system metabolic signaling. The component of the aperlinergic system that affects processes linked to energy metabolism seems to be mediated via the PI3K/Akt and AMPK/eNOS pathways. Both pathways control glucose metabolism, whereas eNOS is implicated in lipolysis and fatty acid oxidation as well.

Apelin stimulates insulin secretion while significantly improving the pancreatic islet cell mass and β-cell insulin in Akita mice, which is a model used for the study of diabetes type 1 [37]. An in vitro study demonstrated that glucose-stimulated insulin secretion may be raised by 30% via a high dose of apelin, while insulin secretion can be strongly suppressed by 50% via decreased apelin doses [35]. This dose-dependent action could be attributed to the presence of different stages of glucose homeostasis. When insulin-sensitizing action diminishes, excess insulin is the only resort to reduce hyperglycemia. There are data pointing to a possible connection between apelin production in adipocytes and the plasma concentrations of insulin [10]. The component of the aperlinergic system that affects processes linked to energy metabolism seems to be mediated via the AMPK/eNOS and PI3K/Akt pathways [10] (Figure 2).

Taken together, the above data show that the apelin/APJ system is capable of promoting glucose utilization through enhancing glucose absorption by adipose tissue and skeletal muscles and modulating insulin resistance and secretion in a dose-dependent manner.

## 3. Apelin in Pregnancy: Fetal Growth, Metabolic Regulation, and Complications

### 3.1. Apelin: Fetal Growth and Metabolic Programming

There were a large number of studies that demonstrated that the apelinergic system is closely linked to mechanisms that promote cell proliferation and inhibit apoptosis, where these processes are obviously vital for fetal growth and development. Of note, during pregnancy, apelin induces placental cell proliferation, angiogenesis, and trophoblast survival, which are processes that play an essential role in placental function and fetal development, especially during the first stages of gestation when cell proliferation represents the main determinant of fetal growth, followed by cell hypertrophy at later stages [38,39].

In vitro studies of syncytiotrophoblast BeWo and cytotrophoblast JEG-3 (human trophoblastic cell lines) showed that apelin induces early placental development by upregulating the APJ and Stat3, ERK1/2, and AMPKα signaling pathways and stimulating the transition to the G2/M phase of the cell cycle [3].

Research into the underlying mechanisms that regulate fetal metabolic programming and growth has evolved into molecular-based studies whereby several mediators, such as adipokines, growth factors, and other molecules, were investigated in depth [40,41,42]. For instance, such studies in animals demonstrated that apelin receptor deficiency during the prenatal period can lead to growth retardation and early embryonic defects, mainly cardiac malformations, and, as a result, to fetal demise [43]. Interestingly, decreased apelin levels in second-trimester amniotic fluid are associated with large-for-gestational-age neonates [44]. The important role of apelin in fetal development is strongly indicated by the increased concentration levels of this adipokine in the umbilical cord blood of the fetus compared with those in maternal plasma [45,46]

Maternal apelin levels are also increased in gestation and, based on animal studies, the origin of this excess appears to be the placenta [46,47]. It has been reported that apelin injections given to mothers are capable of elevating glucose transport through the placenta [46]. The above findings in conjunction imply that apelin is the mediator that augments energy supply to the fetus. On the other hand, there is still a lack of clarity as to how apelin affects intrauterine glucose homeostasis. What is certain is that the optimal control of glucose homeostasis during pregnancy is essential for the development of the fetal–placental unit, as well as for physiological adaptations following birth.

There are, however, few data on the functioning of the apelinergic system in pregnancy. Findings from animal studies demonstrated that both the placenta–fetus interface and various fetal tissues show an expression of the apelinergic system. Maternal undernutrition severely diminishes apelin in the serum of mothers and growth-restricted fetuses while altering the expression of the apelinergic system at the fetal and placental interface. Specifically, serum apelin levels were found to be 30% lower as compared with normal controls, which was a phenomenon that started in early pregnancy and continued till term. Notably, these findings, which were derived from serum studies, are additionally mirrored in reduced placental apelin expression and staining [47]. Moreover, intraperitoneal apelin administered to neonates heightens glucose uptake in the lungs and muscles. Taken together, the above data clearly reveal that glucose homeostasis in fetuses and neonates is regulated by apelin, while, importantly, the adipokine is altered, as stated above, by maternal-undernutrition-induced fetal growth restriction [46].

Since the placenta is the source of apelin, the observed low concentrations of apelin in FGR pregnancies could well be attributable to the placental insufficiency characterizing these mothers. This hypothesis is further supported by the decreased placental apelin staining noted in FGR cases. Interestingly, FGR cases show reduced staining, even compared with preeclampsia cases. While low serum and placental apelin levels could possibly result from reduced placental transcription, with this being tentatively indicated by comparable expression of apelin gene in both growth-restricted and normal placentas, they are more likely to derive from post-transcriptional regulation. They could, on the other hand, be attributed to the smaller placental volume that normally characterize FGR pregnancies, which translates to analogous gene expression that results in a smaller quantity of end products, and thus, lower levels [48]. Interestingly, at the feto–maternal interface of FGR pregnancies, the apelin gene expression is upregulated [46], with this possibly being a compensatory response whereby FGR fetuses enhance their glucose supply and maintain (or improve) their growth.

### 3.2. Apelin and Fetal Hypoxia

Fetal hypoxia, which is the main outcome of placental insufficiency, was shown in animal studies to lead to intracellular fetal liver alterations, that result in insulin resistance and impaired glucose production, which are both key features of diabetes type 2. The latter outcome could also apply to human embryos since it is known that the offspring of pregnancies complicated by placental insufficiency are at a higher risk of progression to type 2 diabetes in adult life [49].

Yet other findings from animal studies point to a notable shift in metabolism to amino acids and lactate as substrates for glucose production instead of lipids. This is probably an adaptive mechanism of the fetal liver in the setting of a hostile hypoxic intrauterine environment; this process maintains the supply of glucose to sensitive vital organs, such as the brain, and shifts energy production from substrates that require high concentrations of oxygen to others utilized under anaerobic conditions. The latter is also likely to be the mechanism of liver steatosis in adults with type 2 diabetes [50].

Studies in adults and babies born with low birth weight for gestational age indicate that resistance to insulin is a characteristic associated with low birth weight, regardless of confounding factors, such as a family history of type 2 diabetes and obesity. Persistent tissue hypoxia and anaerobic cellular metabolism can cause oxidative stress injuries. Since there is a negative correlation in pregnant mothers between apelin and oxidized LDL and HDL cholesterol, this feature could act as an oxidative stress biomarker in gestation [32], while given that apelin improves insulin resistance and exerts antioxidative actions, it could additionally serve as a therapeutic agent that ameliorates these consequences.

### 3.3. Apelin: Cardiovascular Actions and Role in Preeclampsia

The role of apelin goes beyond supporting fetal growth and energy supply since it also vitally regulates gestational hemodynamics, with this being clearly shown in the cardiovascular changes that occur during pregnancy [48]. Apelin, which is a particularly potent inotropic agent, induces endothelium-mediated vasodilatation [51]; indicatively, chronic apelin administration to heart failure patients, which are characterized by low apelin levels, was observed to boost cardiac performance and output [52]. Hence, the decreased maternal apelin levels in FGR pregnancies might theoretically account for the reduced maternal cardiac output observed in these patients [53].

Preeclampsia, which is a complication of pregnancy, is thought to have a common origin to that of FGR, namely, placental insufficiency [54,55]. Furthermore, recent data strongly indicate that an impaired adaptation of the maternal cardiovascular system could be the main mechanism that underlies preeclampsia. With regard to apelin, the findings are conflicting to date, where some studies have reported lower apelin levels in the serum and placental tissue of women with preeclampsia in comparison with healthy pregnant women, and other studies showed increased apelin levels in maternal serum and placental tissue from preeclamptic women compared with controls [48,56,57,58,59]. The latter findings suggest that there could be a severity-dependent association between apelin and preeclampsia. Data regarding the apelin receptor also conflict, with a number of studies showing an increase in placental levels of the receptors in preeclampsia and others depicting a decrease [60,61]. Despite these inconsistencies, there are indications of an association between apelin concentrations and preeclampsia.

The value of apelin in preeclampsia treatment was validated via numerous reports of its pleiotropic effects on the inhibition of inflammation, angiogenesis, vasodilation, and oxidative stress [62,63]. Meanwhile, studies carried out in heart failure patients, as well as the findings of a mouse model of atherosclerosis, showed that apelin reduced blood pressure, acutely so in heart failure subjects. In similar rat models, apelin was found to ameliorate hypertension and proteinuria and to improve pregnancy outcomes. Furthermore, apelin was observed to promote vessel formation and cardiac contractility to protect against aortic inflammation by lowering IL-6 and TNF-α mRNA levels [62].

Remarkably, the administration of apelin has been reported to reverse reduced entire placenta weight and entire fetal weight, while it also increases the embryo survival percentage. Clinical improvement additionally extends to placental function, with this being confirmed by a pathological analysis of placental tissue [62].

With regard to preeclampsia, it was observed that apelin administration in these cases resolves villous edema and irregularity in cell arrangement. Other equally pertinent data that illustrate the beneficial effects of apelin show that it improves the disrupted eNOS/NO signaling pathway [60] and inhibits the activation of oxidative stress [64,65] in both animal models and human studies; it is thus evident that treatment with apelin significantly ameliorates the symptoms of preeclampsia [62]. Rats with preeclampsia present markedly decreased protein and mRNA eNOS placental levels, as well as reduced serum eNOS activity. It is noteworthy that apelin administration results in the enhanced regulation of eNOS protein and mRNA levels, thereby reversing the preeclampsia-induced decrease in both the serum NO content and eNOS activity.

As concerns the part played by oxidative stress in preeclampsia, an examination of plasma from preeclamptic women pointed to reduced endothelium-mediated vasodilation in normal vascular resistance vessels [62]. The fact that abnormal placentation is thought to be the etiology of preeclampsia, while endothelial dysfunction is a major characteristic of this disorder, denotes that the augmentation of NO bioavailability presents itself as a promising approach for preeclampsia prevention and management. NO supplementation with L-arginine together with antioxidant vitamins showed a 40% reduction in the preeclampsia risk factor among women with a history of preeclampsia or with an immediate relative with such a history [62]. Importantly, apelin is known to exert an immediate activation effect on the L-arginine/NO/eNOS pathway [64,65]. A genetic reduction of apelin has been linked to notably elevated COX-2 mRNA, IL-6, and IL-8 expressions, which are directly followed by the secretion of prostaglandins [66]. An interesting study separately evaluated the serum concentrations of the most bioactive apelin peptide fragments, namely, apelin-13 and apelin-36, in preeclampsia patients with the aim of exploring their potential association with perinatal morbidity. The conclusion was that apelin fragments are markers that can effectively differentiate between preeclamptic and healthy pregnancies, although no predictive value for perinatal morbidity could be confirmed [67].

## 4. Therapeutic Potential of Apelin

**Figure 3:**
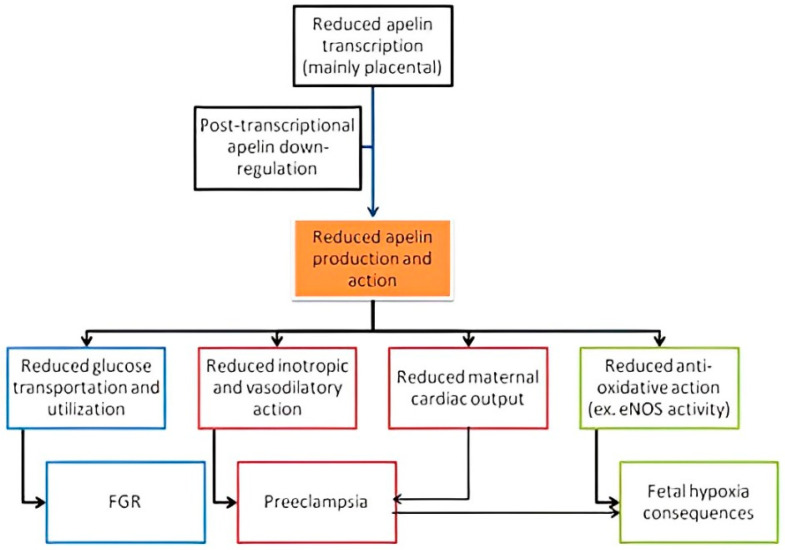
The role of apelin in pregnancy complications. Reduced apelin production is mainly attributed to reduced apelin transcription and post-transcriptional downregulation. The figure illustrates the adverse effects of reduced apelin activity on maternal and fetal health.

The apelinergic system is expressed in a large number of tissues: it contributes to the activation of various intracellular pathways, which leads to the regulation of a range of physiological functions. As a result, apelin plays a significant role in fetal growth disorders, preeclampsia, and obesity-related disorders (Figure 3). Apelin, as mentioned, can regulate glycemic control by increasing the insulin sensitivity and glucose uptake. The therapeutic potential of apelin is limited by its short half-life, but apelin analogs, in particular, apelin-13, have a higher biological half-life and can thus be considered as potential therapeutic agents. Studies in rat models that mimicked diabetes mellitus type 1 (streptozotocin-induced) and diabetes mellitus type 2 (high-fat diet-fed rats) observed that the daily administration of apelin-13 ameliorated insulin sensitivity and decreased plasma glucose levels [68].

Regarding its potential use in the treatment of cardiovascular disorders, apelin can promote angiogenesis and vasodilatation and reduce blood pressure. Studies were conducted in both animals and humans, where the animal studies showed that apelin can potentially be used as a diuretic agent and can protect against arrhythmias and thrombosis as a result of its anti-inflammatory and anti-fibrotic effects. In human studies, when apelin-36, (Pyr(1))apelin-13, and sodium nitroprusside (0.6 nmol/min) were intravenously infused in 24 healthy individuals, apelin caused vasodilation in forearm vessels, which was attenuated by nitric oxide [69].

As mentioned above, apelin’s ability to exert pleiotropic effects on the inhibition of inflammation, angiogenesis, vasodilation, and oxidative stress have been successfully exploited in the treatment of preeclampsia. Apelin administration in these cases resolves villous edema and irregularity in cell arrangement. Furthermore, apelin improves the disrupted eNOS/NO signaling pathway and inhibits the activation of oxidative stress, and hence, ameliorates the symptoms of preeclampsia [62].

## 5. Conclusions

While it is clear that adipokines, such as apelin, exert pleiotropic effects, elucidation of the processes that underlie the molecular and physiological functions of these cytokines has not as yet been understood. The expression of apelin/APJ is seen to occur in numerous tissue types; this decidedly points to a definite role being played by the apelin/APJ system in multiple physiological functions, where the latter include vital processes such as energy metabolism, glucose homeostasis, cardiovascular function, and angiogenesis. On the other hand, it is thought that the alternation of the microenvironmental conditions that promote pathology could generate a modification in the role of apelin.

An important part appears to be played by apelin in obesity-related disorders, diabetes mellitus, fetal growth disorders, and preeclampsia. Given that specifically in the settings of obesity, diabetes, fetal growth restriction, and preeclampsia, changed levels of serum apelin were observed in numerous tissues, it was hypothesized that apelin could act as a treatment target of the above pathologies. Nonetheless, there are as yet limited data supporting this hypothesis despite the fact that apelin administration clearly ameliorates the clinical and histopathological manifestations of these diseases. There is undoubtedly the need for future studies to provide further insight into apelin’s role in intrauterine processes and to precisely determine its value as a prospective therapeutic agent.
